# Endothelin neurotransmitter signalling controls zebrafish social behaviour

**DOI:** 10.1038/s41598-019-39907-7

**Published:** 2019-02-28

**Authors:** Héctor Carreño Gutiérrez, Sarah Colanesi, Ben Cooper, Florian Reichmann, Andrew M. J. Young, Robert N. Kelsh, William H. J. Norton

**Affiliations:** 1 Department of Neuroscience, Psychology and Behaviour, College of Life Sciences, University of Leicester, Leicester, LE1 7RH UK; 2 Department of Biology and Biochemistry and Centre for Regenerative Medicine, University of Bath, Claverton Down, Bath, BA2 7AY UK

## Abstract

The formation of social groups is an adaptive behaviour that can provide protection from predators, improve foraging and facilitate social learning. However, the costs of proximity can include competition for resources, aggression and kleptoparasitism meaning that the decision whether to interact represents a trade-off. Here we show that zebrafish harbouring a mutation in *endothelin receptor aa* (*ednraa*) form less cohesive shoals than wild-types. *ednraa*^−/−^ mutants exhibit heightened aggression and decreased whole-body cortisol levels suggesting that they are dominant. These behavioural changes correlate with a reduction of parvocellular arginine vasopressin (AVP)-positive neurons in the preoptic area, an increase in the size of magnocellular AVP neurons and a higher concentration of 5-HT and dopamine in the brain. Manipulation of AVP or 5-HT signalling can rescue the shoaling phenotype of *ednraa*^−/−^ providing an insight into how the brain controls social interactions.

## Introduction

The distribution of animals within groups represents a trade-off between the costs and benefits of proximity^1^. Closer interaction can improve protection from predators and enhance foraging efficiency, social learning and information transfer, whereas costs include competition for resources, increased aggression and kleptoparasitism^1,2^. The decision whether to join a group is context-dependent, and the form and density of groups can fluctuate permitting individuals to maximise their fitness as circumstances change^3^. Social interaction is a dynamic process that depends upon the size, behaviour and physiology of group members. The balance of costs and benefits to each individual is related to the size of the group and the individual’s spatial position within it^4^. Fish form social groups called shoals (when individuals are loosely associated) or schools if the group exhibits higher synchrony and polarisation^5^.

Social interactions in fish are likely to be controlled by the social decision-making network (SDMN), a group of reciprocally-connected subcortical brain areas^6^ that are important for vertebrate social behaviour. The SDMN interacts with the mesolimbic reward system to assess the salience of stimuli, integrate sensory information and tailor an appropriate behavioural response^7^. Social behaviour is an emergent property of dynamic patterns of activity across the network rather than activity at a single node^8^. Neurotransmitters, neuropeptides and sex steroid hormones can change the weighting of connectivity between nodes making them ideal molecules to regulate sociality^6^.

Endothelins (ET) are 21 amino acid peptide neurotransmitters found in the central nervous system and vasculature of vertebrates^9^. ETs act as both neurotransmitters and neuromodulators in the CNS including the preoptic area of the anterior hypothalamus (POA)^10–12^. They are co-packaged with other neurotransmitters in neurosecretory vesicles and can modulate the release of arginine vasopressin (AVP, called arginine vasotocin (AVT) in fish)^13–15^, and increase dopaminergic neuron activity^16^. In rodents, the ET system also controls blood pressure, sodium homeostasis, water excretion and both central and peripheral nervous system activity^9^. Endothelin-1 (ET-1, one of the three ET isoforms), binds to Endothelin receptor type A (ET-A), a G protein-coupled receptor that is expressed in areas of the rat brain including the locus coeruleus, substantia nigra, nucleus of the solitary tract, ventral tegmental area, periaqueductal grey and the supraoptic and paraventricular nuclei (mammalian homologues of the zebrafish POA)^17–19^. Heterozygous ET-1 knock-out mice are less aggressive and display reduced autonomic response to emotional stress^20^ demonstrating that ETs can modify behaviour. Polymorphisms in both ET and AVP signalling pathway components have been linked to autism spectrum disorder in humans, suggesting possible implications for psychiatric disorders that lead to altered social behaviour^21–25^. This previous research prompted us to further investigate the connection between endothelins and social behaviour in zebrafish. Zebrafish are social animals that display aggression and form dominance-subordinate hierarchies^26–28^. They also school or shoal^5,29^. We therefore hypothesised that a reduction in ET signalling would decrease shoaling in this species. Furthermore, in light of the known interactions between ET, AVP and monoamine signalling^13–16,30,31^, we reasoned that changes to AVP, dopamine and 5-HT neurotransmission could contribute to the behavioural phenotype of *ednraa*^−/−^ mutants.

In this study we have examined *endothelin receptor type aa* (*ednraa*), one of two zebrafish orthologues of the human ET-A gene^32^. We found that *ednraa*^−/−^ mutants are more aggressive and less social than wild-type fish (WT). They exhibit increased inter-individual and nearest neighbour distances compared to WT in a shoaling test. *ednraa*^−/−^ also have less whole-body cortisol than WT suggesting that they are socially dominant, in agreement with studies showing higher cortisol levels in subordinate WT zebrafish^33^. There are fewer *arginine vasopressin* (*avp*)-expressing cells in the POA, and the remaining cells are larger than in WT. There is also a heightened levels of dopamine and serotonin (5-hydroxytryptamine, 5-HT) in the brain of *ednraa*^−/−^. Acute treatment with either the 5-HT1A receptor partial agonist buspirone or AVP can rescue the mutant social phenotype, providing a mechanistic insight into how ET signalling modifies vertebrate social behaviour.

## Results

### Reduction of *ednraa* decreases zebrafish social behaviour

**Figure 1:**
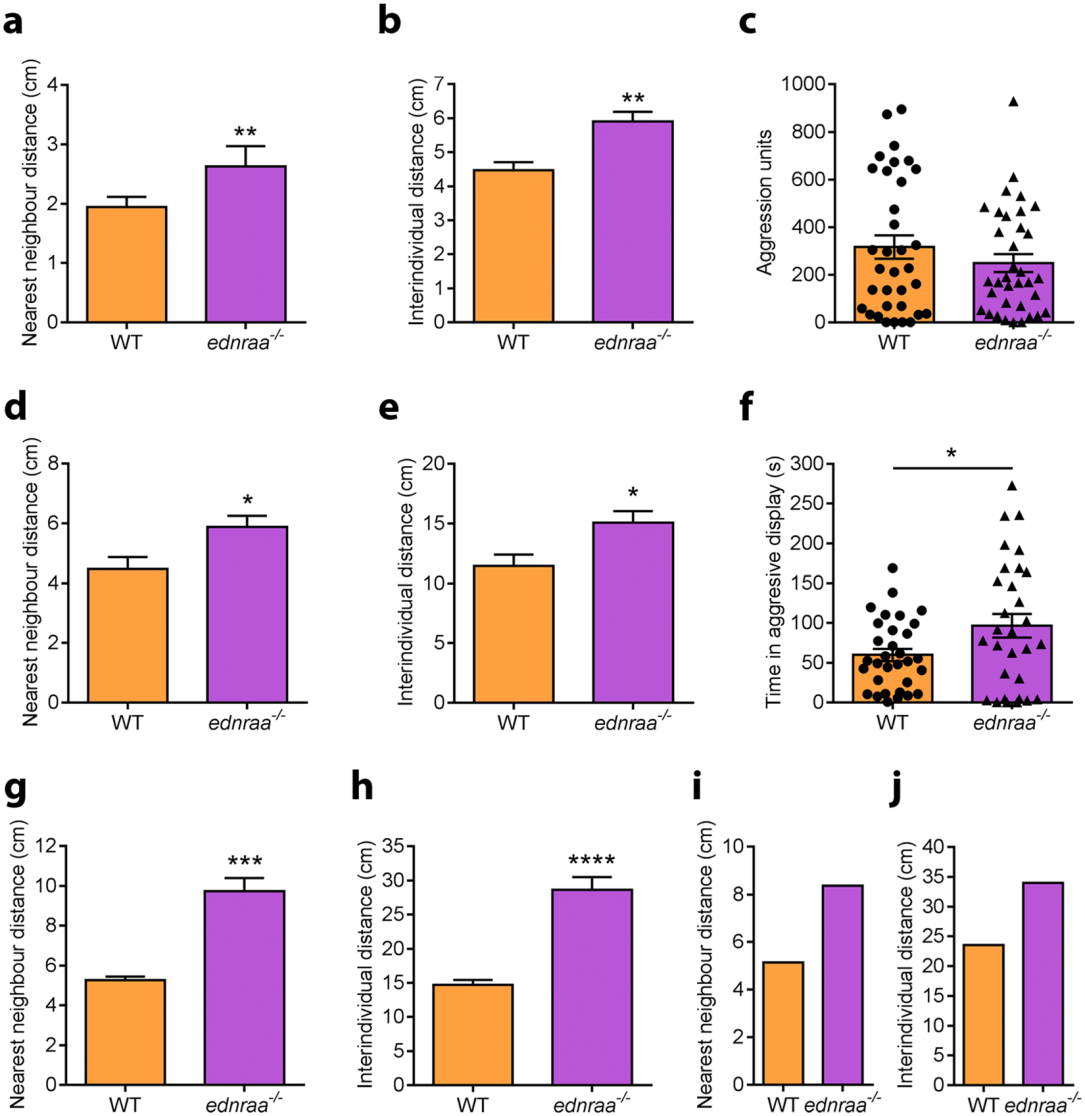
Alterations to social behaviour in *ednraa*^−/−^. (**a)** Shoaling test. Increase in nearest neighbour distance in one-month old *ednraa*^−/−^ compared to WT. **(b)** Shoaling test. Increase in inter-individual distance in 1-month old *ednraa*^−/−^ compared to WT. **(c)** No difference in mirror-induced aggression levels between 1-month old *ednraa*^−/−^ and WT quantified using Viewpoint ZebraLab software. **(d)** Increase in nearest neighbour distance in adult *ednraa*^−/−^ compared to WT. **(e)** Increase in inter-individual distance in adult *ednraa*^−/−^ compared to WT. **(f)** Adult *ednraa*^−/−^ are more aggressive than WT in a mirror-induced aggression test. Manual quantification of data. **(g)** Shoaling test. Increase in nearest neighbour distance and **(h)** Shoaling test. Inter-individual distance between mutants and WT tested in a large tank. **(i)** Shoaling test. Average nearest neighbour distance and **(j)** Shoaling test. Inter-individual distance of 16 fish in a large tank. *p < 0.05, **p < 0.01, ***p < 0.001, ****p < 0.0001. Each dot represents one fish.

**Figure 2:**
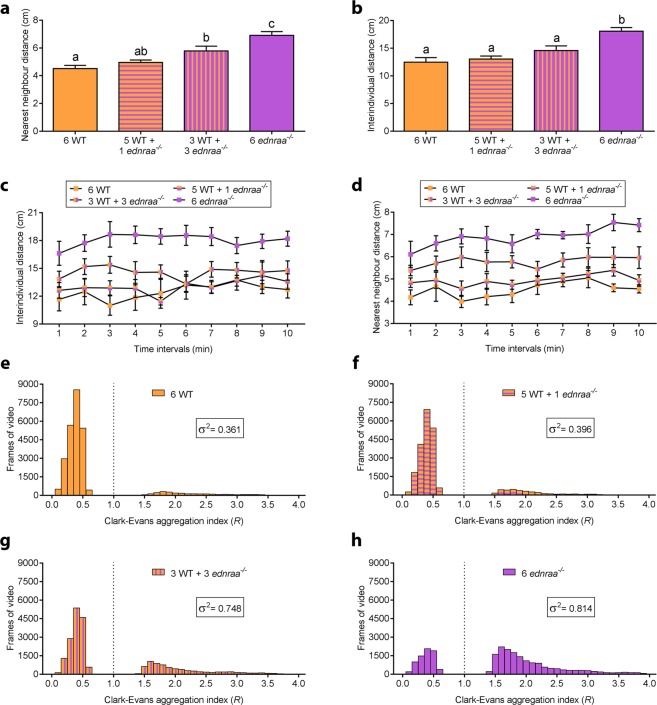
Social spacing in groups of mixed genotypes. (**a)** Shoaling test. Mixed groups of *ednraa*^−/−^ and WT (3:3) display an intermediate nearest neighbour distance and **(b)** inter-individual distance. **(c)** Average nearest neighbour and **(d)** inter-individual distance of the same fish as in **a** and **b** represented over time. **(e**–**h)** Shoaling test. Histograms showing the increase in the Clark-Evans aggregation index *R* as the number of *ednraa*^−/−^ in the group of 6 fish increases. The variance of the data (inset) also increases with the number of mutants present in the group. Only frames in which aggregation or repulsion were significant (p < 0.05) are shown here.

During routine stock keeping, we noticed that zebrafish *endothelin receptor aa* (*ednraa*^−/−^) mutants were less social and more aggressive than wild-type (WT) fish. We first measured the distance between one month-old juvenile zebrafish in a shoaling test. At this stage *ednraa*^−/−^ had a larger nearest neighbour distance (Fig. 1a, t-test: t_(10)_ = 4.417, p = 0.0013; n = 6 groups of 6 fish each genotype) and inter-individual distance (Fig. 1b, t-test: t_(10)_ = 3.913, p = 0.0029; n = 6 groups of 6 fish each genotype) compared to WT. There was no difference in mirror-induced aggression between genotypes (Fig. 1c, Mann Whitney test: U = 538, p = 0.49; n = 34 WT, n = 35 *ednraa*^−/−^). The decrease in social behaviour was maintained at adult stages. In a shoaling test adult *ednraa*^−/−^ exhibited a larger nearest neighbour distance (Fig. 1d, t-test: t_(16)_ = 2.545, p = 0.022; n = 9 groups of 6 fish each genotype) and inter-individual distance (Fig. 1e, t-test: t_(16)_ = 2.648, p = 0.017; n = 9 groups of 6 fish each genotype) when recorded in groups of 6 fish in a medium-sized tank (43 × 22 cm). Adult *ednraa*^−/−^ also spent more time being aggressive than WT in a mirror-induced aggression test (Fig. 1f, t-test (Welch): t_(43.85)_ = 2.189, p = 0.034; n = 30 WTs, n = 32 *ednraa*^−/−^). The size of the tank used to measure behaviour might restrict the mutant from fully expressing its behavioural phenotype. We investigated this idea by repeating the shoaling test using a larger tank (80 × 40 cm). In this setup, the distance between *ednraa*^−/−^ was even larger whereas WTs did not alter their social interaction (Fig. 1g,h; nearest neighbour distance, t-test (Welch): t_(5.695)_ = 6.518, p = 0.0008; inter-individual distance, t-test: t_(10)_ = 6.885, p < 0.0001; n = 6 groups of 6 fish each genotype). The decreased social interaction was also seen when a larger group of fish (n = 16 individuals) was examined (Fig. 1i,j and Film 1,2). We used the Clark-Evans index to examine attraction and repulsion between zebrafish in a shoal^34^. The Clark-Evans index is the ratio of the mean nearest neighbour distance of the fish in a group to the mean nearest neighbour distance in a random Poisson distribution^34^. *R* > 1 indicates a greater nearest neighbour distance than random distribution (repulsion), and *R* < 1 indicates a smaller nearest neighbour distance than random distribution (aggregation). R was calculated for each frame of each video, and frames showing either significant attraction or significant repulsion were recorded (see Methods). *ednraa*^−/−^ fish display a higher Clark-Evans index *R* than WT (Fig. S1a,b). A shoal of 16 WT fish had a mean *R* of 0.61, whilst a shoal of 16 *ednraa*^−/−^ had a significantly higher mean *R* of 1.59 across all frames of the video (Fig. S1a,b, t-test: t (1, 28) = 208.6, p < 0.0001) suggesting that they repulse each other more than WT. We next tested mixed groups of WT and *ednraa*^−/−^ in a shoaling test using a medium-sized tank (Fig. 2). Groups containing only WT or mutants showed a significant difference in social behaviour in agreement with our previous results. However, heterogeneous groups of three WT and three mutants gave rise to an intermediate difference in nearest neighbour distance (Fig. 2a,c; one-way ANOVA followed by Tukey’s post hoc: F (3, 21) = 14.02, p < 0.0001) and inter-individual distance (Fig. 2b,d; one-way ANOVA followed by Tukey’s post hoc: F (3, 21) = 11.13, p = 0.0001; n = 6 groups of 6 fish each). As the proportion of *ednraa*^−/−^ in a shoal of 6 fish increased, the number of frames showing significant attraction steadily decreased and the number of frames showing significant repulsion increased (Figs 2e–h and S2a,b). Furthermore, the greater the number of *ednraa*^−/−^ animals in the shoal the greater the variance of *R* (Fig. 2e–h), indicating that the shoal has a wider range of potential spatial configurations when more mutants are present. Shoals containing 6 *ednraa*^−/−^ fish showed significantly less aggregation than shoals of 6 WT (Figure S2a, one-way ANOVA followed by Tukey’s post hoc: F (3, 20) = 3.89, p = 0.018). Shoals of 6 *ednraa*^−/−^ fish also showed more instances of significant repulsion than shoals containing 6 WT, 5 WT + 1 *ednraa*^−/−^, and 3 WT + 3 *ednraa*^−/−^ (Fig. S2b, one-way ANOVA followed by Tukey’s post hoc: F (3, 20) = 15.2, p < 0.0001, 6 *ednraa*^−/−^ vs 3 *ednraa*^−/−^ + 3 WT p = 0.004; 6 *ednraa*^−/−^ vs 1 *ednraa*^−/−^ 5 WT p < 0.001; 6 *ednraa*^−/−^ vs 6 WT p < 0.001).

### Social preference is impaired in *ednraa*^−/−^

**Figure 3:**
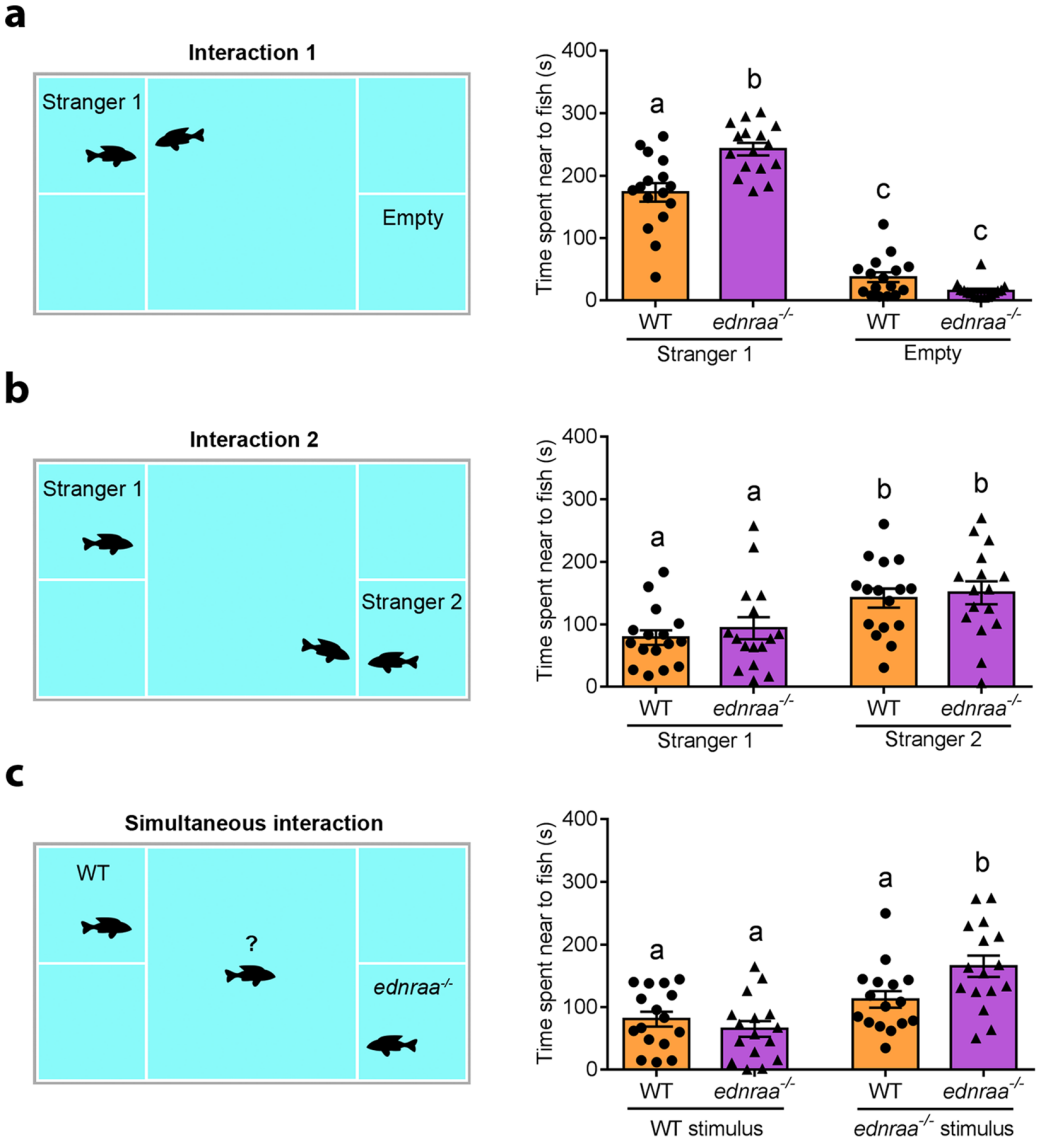
Social interaction and discrimination in the social preference test. (**a)** Social preference. WT and *ednraa*^−/−^ spend more time interacting with stranger 1 than in the empty area and *ednraa*^−/−^ spend more time interacting with the stimulus fish than WT. **(b)** Social novelty. Both genotypes switch preference to interact with a second WT stimulus. **(c)** Social discrimination. When both *ednraa*^−/−^ and WT are used as a stimulus, *ednraa*^−/−^ prefer to interact with *ednraa*^−/−^ whereas WT displays no preference. Each dot represents one fish. Letters not shared in common between or amongst groups indicate significant differences from Tukey’s (significant interaction) or Sidak’s (non-significant interaction) post hoc comparison after two-way ANOVA, p < 0.05.

We used the social preference test to investigate social interaction and discrimination at the individual level. We placed a focal WT or *ednraa*^−/−^ fish in the centre of the social preference tank and recorded its interaction with an unfamiliar female WT stimulus fish (stranger 1). Both WT and *ednraa*^−/−^ spent more time in the quadrant next to stranger 1 than in the empty control area (Fig. 3a, WT stranger 1 vs empty: *p* < 0.0001, *ednraa*^−/−^ stranger 1 vs empty: *p* < 0.0001). Surprisingly, we found that mutants spent significantly more time interacting with stranger 1 than WT (Fig. 3a, total time spent near stimulus fish; WT vs *ednraa*^−/−^, p < 0.0001; two-way ANOVA followed by Tukey’s post hoc, genotype factor: F (1, 60) = 5.747, p = 0.019, stranger factor: F (1, 60) = 336, p < 0.0001, interaction genotype × stranger: F (1, 60) = 21.16, p < 0.0001; n = 16 WT, n = 16 mutant). The interaction with stranger 1 included aggression. WT fish spent 38% of the social interaction time in aggressive display (mean ± SEM = 68 ± 15 s), whereas *ednraa*^−/−^ fish spent 59% of time (mean ± SEM = 147 ± 22 s) being aggressive (manual quantification of aggression of WT vs *ednraa*^−/−^; t-test: t_(30)_ = 2.947, p = 0.0062; n = 16 WT, n = 16 mutant). This suggests that the social preference test has a weak agonistic component that may not occur in the shoaling test, perhaps because stimulus fish are unable to swim away from their opponents. We assessed preference for social novelty by introducing a second unfamiliar female stimulus (stranger 2) into the same tank. Both WT and *ednraa*^−/−^ switched their preference and spent more time in the quadrant close to stranger 2 with no difference between genotypes (Fig. 3b, total time spent near stranger fish; WT stranger 1 vs stranger 2, p = 0.013; *ednraa*^−/−^ stranger 1 vs stranger 2, p = 0.028; WT vs *ednraa*^−/−^ stranger 1, p = 0. 75; WT vs *ednraa*^−/−^ stranger 2, p = 0.91; two-way ANOVA followed by Sidak’s post hoc, genotype factor: F (1, 60) = 0.5709, p = 0.45, stranger factor: F (1, 60) = 14.26, p = 0004, interaction genotype × stranger: F (1, 60) = 0.0423, p = 0. 84; n = 16 WT, n = 16 mutant).

To investigate the influence of the stimulus fish’s genotype on the results of the social preference test we allowed focal fish to interact with an unfamiliar WT or *ednraa*^−/−^ fish presented simultaneously. WT focal fish spent a similar amount of time in the quadrants close to fish of both genotypes whereas *ednraa*^−/−^ spent more time close to mutants rather than WT (Fig. 3c, total time spent near stimulus fish; focal WT: p = 0.3806, focal *ednraa*^−/−^: p < 0.0001; two-way ANOVA followed by Tukey’s post hoc, genotype (focal fish) factor: F (1, 60) = 1.824, p = 0.1819, genotype (stimulus fish) factor: F (1, 60) = 22.61, p < 0.0001, interaction genotype × genotype: F (1, 60) = 6.138, p = 0.0161; n = 16 WT, n = 16 mutant). In agreement with our first experiment, there was an aggressive component to the interaction with the *ednraa*^−/−^ stimulus zebrafish (focal WT: mean ± SEM = 9 ± 4 s, compared to focal *ednraa*^−/−^: mean ± SEM = 76 ± 15 s aggression; manual quantification of aggression of WT vs *ednraa*^−/−^; t-test (Welch): t_(16.99)_ = 4.237, p = 0.0006; n = 16 WT, n = 16 mutant). This suggests that when a conspecific is in close proximity to *ednraa*^−/−^ (i.e. less than the usual nearest neighbour distance) heightened aggression may lead to decreased social interactions.

### Locomotion, anxiety-like behaviour and novel object interaction are not altered in *ednraa*^−/−^ zebrafish

**Figure 4:**
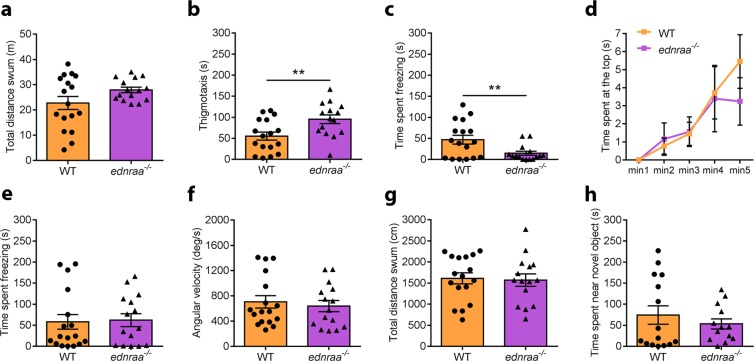
Non-social behaviour of *ednraa*^−/−^. (**a)** Open field test. *ednraa*^−/−^ and WT swim a similar distance in the open field test. In this test *ednraa*^−/−^ display **(b)** increased thigmotaxis and **(c)** decreased freezing compared to WT. **(d)** Novel tank test. *ednraa*^−/−^ spend a similar amount of time in the top third of a novel tank as WT. **(e)** Freezing, **(f)** angular velocity and **(g)** distance swum in the novel tank test are also similar in WT and *ednraa*^−/−^. **(h)** Time spent near to a novel object is similar in WT and *ednraa*^−/−^. **p < 0.01. Each dot represents one fish.

We evaluated whether the social phenotype of *ednraa*^−/−^ fish was accompanied by changes to other behaviours by measuring locomotion, anxiety-like behaviour and novel object interaction. Both WT and *ednraa*^−/−^ showed similar locomotion in the open field test (Fig. 4a, t-test (Welch): t_(21.71)_ = 1.844, p = 0.0789; n = 15 WT, n = 17 *ednraa*^−/−^) whereas *ednraa*^−/−^ displayed a small increase in thigmotaxis (time spent swimming within 2 cm of the tank walls; Fig. 4b, t-test: t_(30)_ = 2.863, p = 0.0076) which could be interpreted as anxiety-like behaviour or stereotypy^35^. Mutants also spent a smaller amount of time freezing (Fig. 4c, t-test (Welch): t_(22.22)_ = 2.833, p = 0.0096). We further investigated anxiety-like behaviour in the novel tank diving test^36^. Fish of both genotypes spent a similar amount of time in the top third of the tank (Fig. 4d, Mann-Whitney test: U = 108, p = 0.4525; n = 15 WT, n = 17 *ednraa*^−/−^) and there were no differences in freezing (Fig. 4e, t-test: t_(30)_ = 0.1762, p = 0.8613), mean angular velocity (Fig. 4f, t-test: t_(30)_ = 0.5007, p = 0.6202) or the distance swum (Fig. 4g, t-test: t_(30)_ = 0.2108, p = 0.8345) suggesting that *ednraa*^−/−^ have no anxiety-like phenotype. *ednraa*^−/−^ also showed no difference compared to WT when interacting with a novel object (Fig. 4h, t_(21.56)_ = 0.8498, p = 0.4048; n = 15 WT, n = 13 *ednraa*^−/−^) in the novel-object test. Together, these results show that *ednraa*^−/−^ display a decrease in shoaling that cannot be accounted for by changes in locomotion or anxiety-like behaviour.

### Altered distribution of arginine vasopressin neurons in the preoptic area of *ednraa*^−/−^

**Figure 5:**
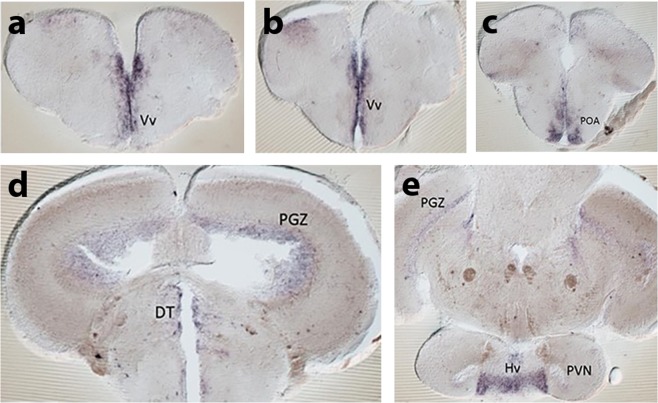
Expression of *ednraa* in the adult zebrafish brain. (**a**–**e)** *In situ* hybridisation showing *ednraa* gene expression in the ventral zone of the ventral telencephalon (Vv), the preoptic area (POA), dorsal thalamus (DT), periaqueductal grey (PGZ), ventral hypothalamus (Hv) and periventricular nucleus of the hypothalamus (PVN).

**Figure 6:**
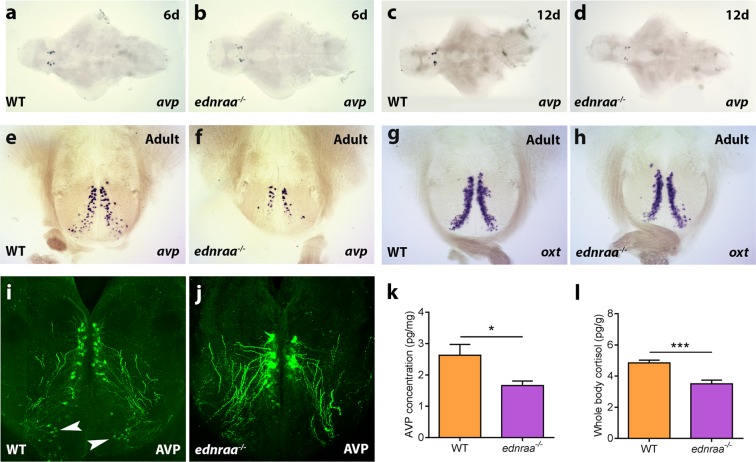
Altered distribution of arginine vasopressin neurons in *ednraa*^−/−^. *In situ* hybridisation showing that expression of *arginine vasopressin* (*avp*) is similar in the brain of WT (**a**,**c**,**e**) and *ednraa*^−/−^ (**b**,**d**,**f**) at 6 days **(a**,**b)** and 12 days **(c**,**d)**. **(e**,**f)** Reduced expression of *avp* in the ventral parvocellular preoptic area of *ednraa*^−/−^ of adult fish. **(g**,**h)** *In situ* hybridisation showing that *oxytocin* (*oxt*) expression is similar in WT and *ednraa*^−/−^ adult fish. **(i**,**j)** Anti-AVP antibody staining shows reduced labelling in the parvocellular preoptic area of *ednraa*^−/−^ (**j**) compared to WT (**i**) (arrowheads). Dorsal magnocellular neurons have a larger cell body and thicker projections. **(k)** Reduced levels of AVP in the brain of *ednraa*^−/−^ compared to WT. **(l)** Decreased whole-body cortisol levels in *ednraa*^−/−^ compared to WT. *p < 0.05, ***p < 0.001.

**Figure 7:**
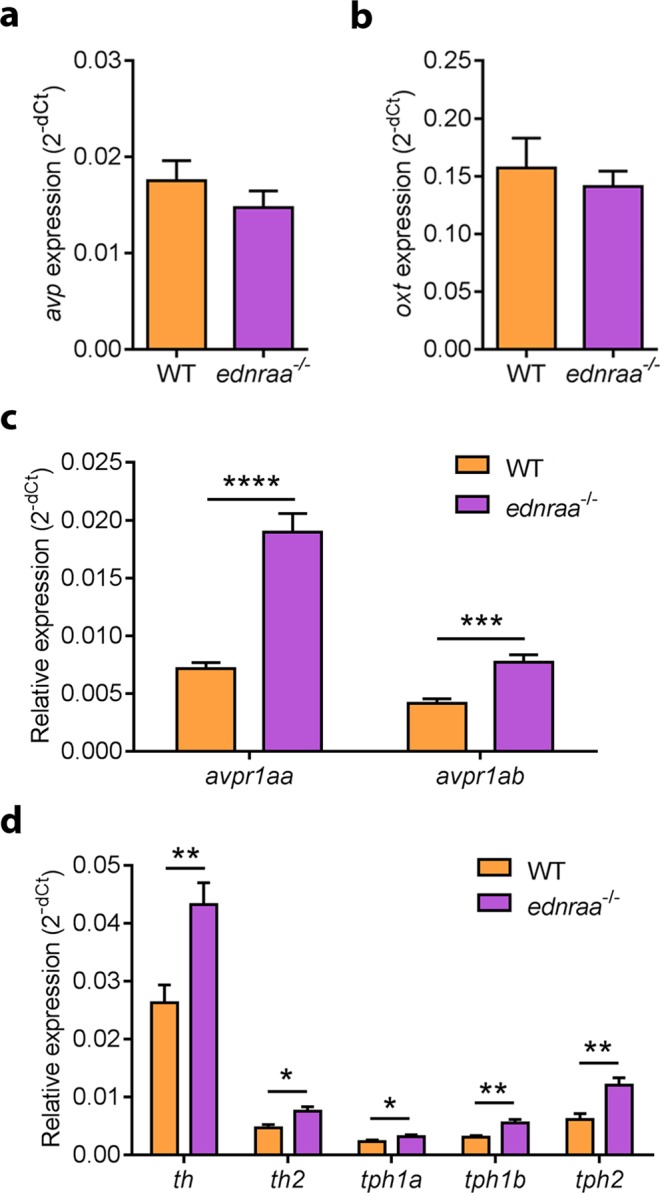
Expression of AVP and monoamine signalling pathway genes in *ednraa*^−/−^. qPCR data showing similar expression of **(a)** *arginine vasopressin* (*avp*) and **(b)** *oxytocin* (*oxt*) in *ednraa*^−/−^ brains compared to WT. Increased expression of **(c)** *arginine vasopressin receptor 1aa* (*avpr1aa*) and *arginine vasopressin receptor 1ab* (*avpr1ab*) in *ednraa*^−/−^ brains compared to WT. **(d)** Increased expression of *tyrosine hydroxylase 1* (*th*), *tyrosine hydroxylase 2* (*th2*), *tryptophan hydroxylase 1a* (*tph1a*), *tryptophan hydroxylase 1b* (*tph1b*) and *tryptophan hydroxylase 2* (*tph2*) in *ednraa*^−/−^ compared to WT. Multiple t-tests with Holm-Sidak multiple comparisons correction. *p < 0.05, **p < 0.01, ***p < 0.001, ****p < 0.0001.

In mammals, ET-A activation releases arginine vasopressin (AVP) from magnocellular neurons of the supraoptic and paraventricular nuclei^13^, mammalian homologues of the zebrafish preoptic area (POA). Differences in the size and position of POA AVP neurons correlate with aggression and social dominance in zebrafish^37^. This makes AVP an ideal candidate to underpin the behavioural phenotype of *ednraa*^−/−^. We first examined the expression of *ednraa* mRNA in the adult zebrafish brain (Fig. 5a-e). *ednraa* mRNA was expressed in the ventral zone of the ventral telencephalon, the POA, dorsal thalamus, periaqueductal grey, ventral hypothalamus and periventricular nucleus of the hypothalamus (PVN), areas of the brain that are important for social behaviour. We next examined expression of the genes coding for AVP and OXT by *in situ* hybridisation. At 6 and 12 days post-fertilisation *arginine vasopressin* (*avp*) mRNA expression in the POA was similar in WT and *ednraa*^−/−^ mutant zebrafish (Fig. 6a–d). However, in the adult brain there were fewer *arginine vasopressin* (*avp*) mRNA-expressing cells in ventral part of the POA in mutant fish (Fig. 6e,f; WT: mean ± SEM = 79 ± 5 cells; *ednraa*^−/−^: mean ± SEM = 54 ± 4 cells; WT vs *ednraa*^−/−^, t-test: t_(9)_ = 4.022, p = 0.0030; n = 5 WT, n = 6 *ednraa*^−/−^). The AVP-positive neurons that were present in the POA of mutants were the more dorsal magnocellular population that had a larger diameter than those found in WT (WT mean diameter: 8.75 ± 0.44 µm; *ednraa*^−/−^ mean diameter: 11.65 ± 0.39; t-test: t_(4)_ = 4.987, p = 0.0076). This suggests that it is the parvocellular population of AVP neurons that show reduced *avp* mRNA expression in mutants. However, there was a similar number of *oxytocin* (*oxt*) mRNA-expressing cells in both genotypes ruling out a global disorganisation of the POA (Fig. 6g,h, WT: mean ± SEM = 279 ± 8 cells; *ednraa*^−/−^: mean ± SEM = 278 ± 19 cells; WT vs *ednraa*^−/−^, t-test: t_(9)_ = 0.0294, p = 0.9772; n = 6 WT, n = 5 *ednraa*^−/−^). We confirmed the reduction of parvocellular AVP-positive neurons by labelling with an anti-AVP antibody^38^. In *ednraa*^−/−^, the dorsal magnocellular AVP neurons had a larger cell body and thicker projections than those in WT (Fig. 6i,j). In the parvocellular POA there were fewer or no AVP-positive neurons and a loss of the associated projections in mutants (Fig. 6i,j). We measured the concentration of AVP in the brain by enzyme-linked immunosorbent assay (ELISA) and found a 37% reduction of AVP in *ednraa*^−/−^ compared to WT (Fig. 6k, t-test (Welch): t_(11.82)_ = 2.57, p = 0.0249; n = 9 WT, n = 7 *ednraa*^−/−^). RT-qPCR analysis demonstrated that the genes coding for *avp* and *oxt* were expressed similarly in both genotypes (Fig. 7a,b; *avp*, t-test: t_(14)_ = 1.04, p = 0.3158; *oxt*, t-test: t_(14)_ = 0.5391, p = 0.5983; n = 8 each). However, there was a strong increase in expression of mRNA for the AVP receptor-encoding genes *avpr1aa* and *avpr1ab* in mutants (Fig. 7c; *avpr1aa*, t-test: t_(14)_ = 6.985, p < 0.0001; *avpr1ab*, t-test: t_(14)_ = 4.781, p = 0.0003) perhaps to compensate for the decreased level of neurotransmitter. *ednraa*^−/−^ fish also had a reduction in basal whole-body cortisol levels suggesting that the hypothalamus-pituitary interrenal axis (the teleost homologue of the hypothalamus-pituitary adrenal axis) is less active (Fig. 6l, t-test: t_(21)_ = 4.368, p = 0.0003; n = 11 WT, n = 12 mutant). Taken together, the altered distribution of POA AVP neurons and the reduction of both AVP and cortisol suggest that *ednraa*^−/−^ display a socially dominant phenotype^33,37^.

### Increased monoamine content in *ednraa*^−/−^ zebrafish

**Figure 8:**
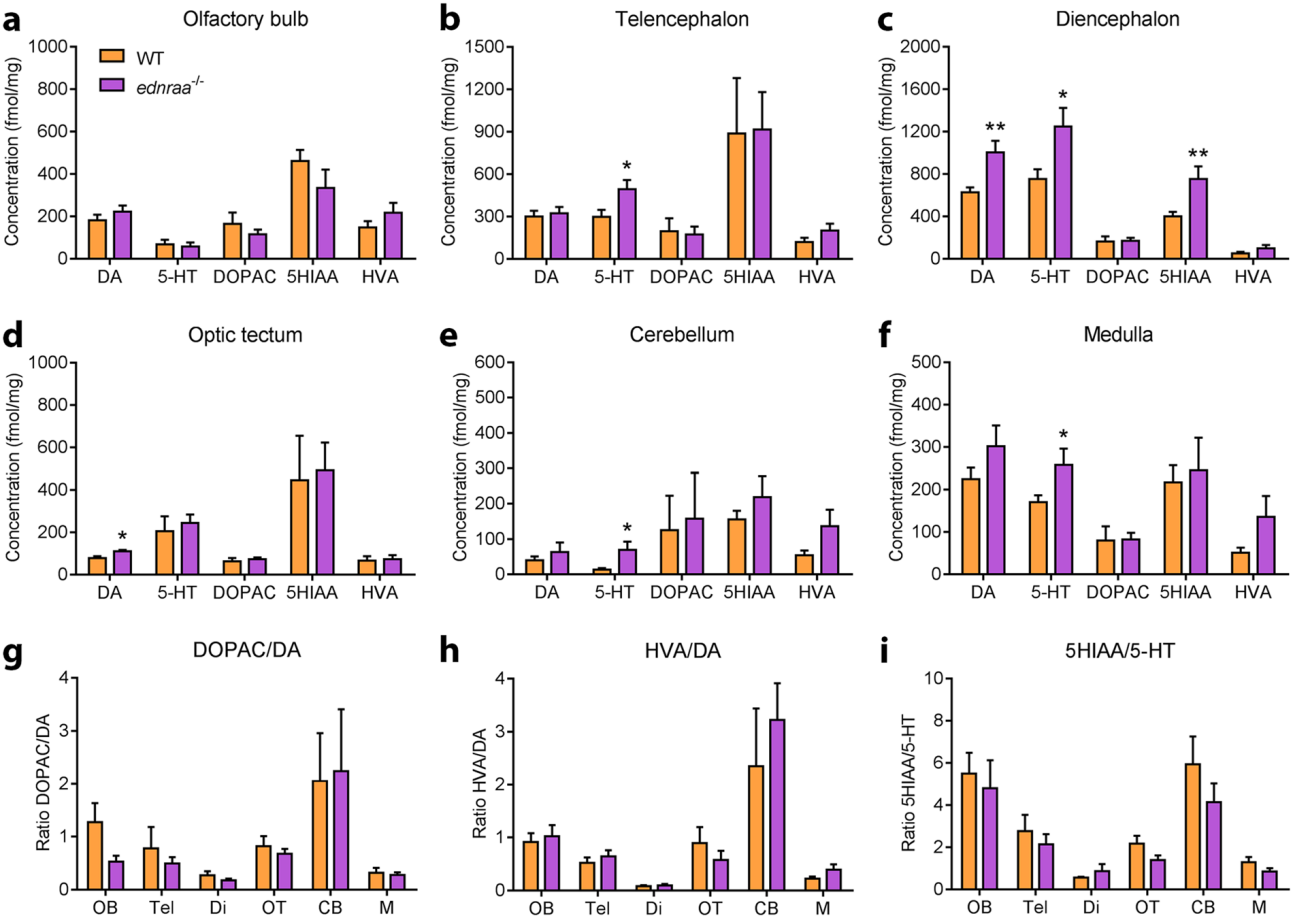
Increased basal levels of 5-HT and dopamine in *ednraa*^−/−^. (**a)** There are no differences in the olfactory bulb. 5-HT levels are increased in the **(b)** telencephalon, **(c)** diencephalon, **(d)** cerebellum and **(f)** medulla of *ednraa*^−/−^ compared to WT. Dopamine levels are significantly higher in the **(c)** diencephalon and **(e)** optic tectum of *ednraa*^−/−^ compared to WT. **(c)** 5HIAA is increased in the diencephalon of *ednraa*^−/−^. **(g–i)** There are no differences in the utilisation ratio of dopamine and 5-HT in *ednraa*^−/−^ compared to WT. n = 9 each genotype. *p < 0.05, **p < 0.01. OB: olfactory bulb, Tel: telencephalon, Di: diencephalon, OT: optic tectum, CB: cerebellum, M: medulla.

ETs can modulate the production and release of both 5-HT and dopamine^16,30,31^. We used high pressure liquid chromatography (HPLC) to measure the basal levels of dopamine, 5-HT and their metabolites (5-hydroxyindoleacetic acid (5HIAA), dihydroxyphenylacetic acid (DOPAC) and homovanillic acid (HVA)) in the brain. 5-HT levels were increased in the telencephalon (Fig. 8b, t-test: t_(15)_ = 2.38, p = 0.0306), diencephalon (Fig. 8c, t-test: t_(14)_ = 2.66, p = 0.0186), cerebellum (Fig. 8e, t-test: t_(15)_ = 2.42, p = 0.0286), and medulla (Fig. 8f, t-test: t_(13)_ = 2.21, p = 0.0455) of *ednraa*^−/−^ compared to WT. 5HIAA was also increased in the diencephalon of mutants (Fig. 8c, t-test: t_(14)_ = 3.11, p = 0.0075). We found a significant increase of dopamine in the diencephalon (Fig. 8c, t-test: t_(14)_ = 3.46, p = 0.0037) and optic tectum (Fig. 8d, t-test: t_(14)_ = 2.68, p = 0.0177; multiple t-tests with Holm-Sidak multiple comparisons correction, n = 7–9 brain regions each genotype) of *ednraa*^−/−^. We also calculated the utilisation ratio of metabolite to neurotransmitter. HPLC measures the sum basal level of analytes in the brain regardless of whether they are stored in synaptic vesicles or have been released into the cleft. Neurotransmitters are broken down to their metabolites upon release. This means that the utilisation ratio gives an approximation of activity for the neurotransmitter being measured^39^. There was no difference in utilisation ratios of dopamine to DOPAC and HVA (Fig. 8g,h) or 5-HT to 5HIAA (Fig. 8i). The augmented levels of dopamine and 5-HT in several regions of the mutant brain could be explained by increased production of these neurotransmitters. We measured the expression of genes coding for the dopamine and 5-HT synthesis enzymes Tyrosine hydroxylase and Tryptophan hydroxylase (Fig. 7d). There was a significantly higher expression of *tyrosine hydroxylase 1* (t-test: t_(14)_ = 3.502, p = 0.0035), *tyrosine hydroxylase 2* (t_(14)_ = 2.965, p = 0.0102), *tryptophan hydroxylase 1a* (t_(14)_ = 2.274, p = 0.0392), *tryptophan hydroxylase 1b* (t_(14)_ = 3.590, *p* = 0.0030) and *tryptophan hydroxylase* 2 (t_(14)_ = 3.641, p = 0.0027; multiple t-tests with Holm-Sidak multiple comparisons correction, n = 7-9 brain regions each genotype) in mutants compared to WT. Dysregulation of 5-HT and dopaminergic signalling therefore represents another mechanism by which social behaviour could be altered in *ednraa*^−/−^.

### Activation of AVP or 5-HT signalling rescues the social phenotype of *ednraa*^−/−^

**Figure 9:**
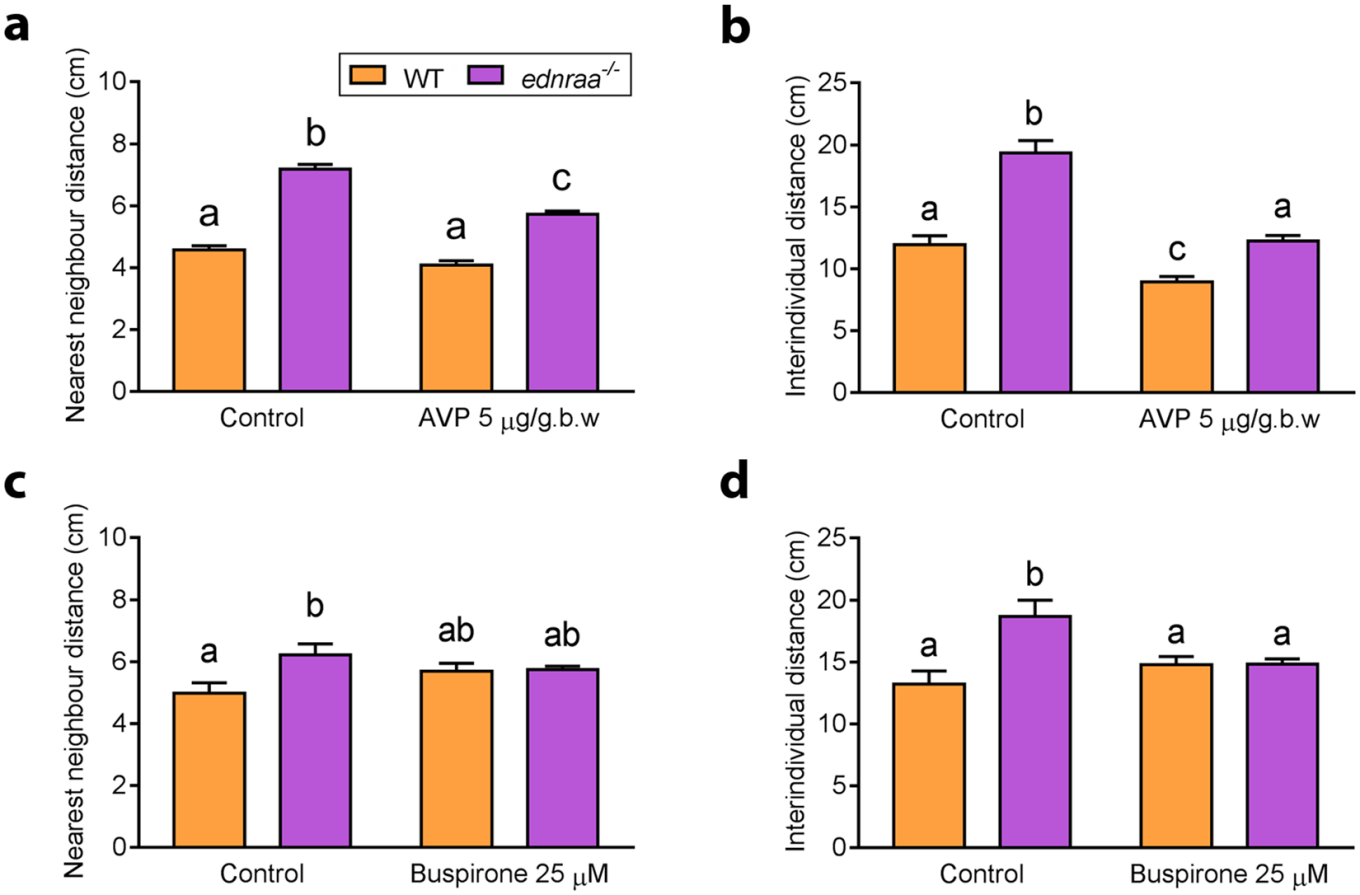
Treatment with AVP or buspirone rescues the shoaling phenotype of *ednraa*^−/−^. (**a**,**b)** Injection of AVP reduces the nearest neighbour- and inter-individual distances in both WT and *ednraa*^−/−^. However, *ednraa*^−/−^ still exhibit increased NND and IID compared to WT. **(c**,**d)** Acute immersion in buspirone reduces the nearest neighbour- and inter-individual distances in *ednraa*^−/−^, rescuing their shoaling phenotype. Letters not shared in common between or amongst groups indicate significant differences from Tukey’s post hoc comparisons after two-way ANOVA, p < 0.05.

Loss of *ednraa* function leads to changes in AVP and monoamine neurotransmitter levels. We investigated the connection between neurobiology and social behaviour by treating WT and mutant zebrafish with either AVP or buspirone hydrochloride, a 5-HT1A receptor partial agonist^40^. Intraperitoneal injection of AVP has already been shown to increase social preference in zebrafish^41^. We treated both genotypes with 5 µg/gbw AVP and measured behaviour in a shoaling test. AVP decreased the nearest neighbour distance in mutant fish but not WT (Fig. 9a, two-way ANOVA followed by Tukey’s post hoc, genotype factor: F (1, 20) = 240.3, p < 0.0001; treatment factor: F (1, 20) = 51.07, p < 0.0001; genotype × treatment interaction: F (1, 20) = 12.61, p = 0.0020; n = 6 groups of 6 fish each genotype) although there was still a difference in nearest neighbour distance between genotypes (p < 0.0001). AVP also decreased the inter-individual distance in both the WT and the mutants, but had a stronger effect in *ednraa*^−/−^, reducing the difference between genotypes (Fig. 9b, two-way ANOVA followed by Tukey’s post hoc, genotype factor: F (1, 20) = 60.56, p < 0.0001; treatment factor: F (1, 20) = 54.06, p < 0.0001; genotype × treatment interaction: F (1, 20) = 8.858, p = 0.0075). In fact, AVP-treated mutants showed a similar inter-individual distance as saline-injected WT fish. However, there was still a significant difference in inter-individual distance between genotypes after AVP injection (p = 0.0139) suggesting that AVP had not fully rescued this behaviour at the dose that we applied.

To investigate the function of 5-HT signalling in the mutant brain we immersed zebrafish in buspirone, a partial 5-HT1AR agonist that decreases 5HT signalling^42^. Treatment with buspirone had no effect on WT but decreased both the nearest neighbour distance (Fig. 9c, two-way ANOVA followed by Tukey’s post hoc, genotype factor: F (1, 19) = 5.544, p = 0.0294; treatment factor: F (1, 19) = 0.181, p = 0.6753; genotype × treatment interaction: F (1, 19) = 4.554, p = 0.0461) and the inter-individual distance (Fig. 9d, two-way ANOVA followed by Tukey’s post hoc, genotype factor: F (1, 19) = 8.532, p = 0.0088; treatment factor: F (1, 19) = 1.427, p = 0.2469; genotype × treatment interaction: F (1, 19) = 8.335, p = 0.0094) of *ednraa*^−/−^ to WT levels showing that mutants are more sensitive to 5-HT manipulation that WT. This demonstrates that both AVP and 5-HT signalling act downstream of *ednraa* to increase the cohesion of groups of mutant fish.

## Discussion

We have demonstrated that Endothelin (ET) neurotransmitter signalling underpins key elements of zebrafish social behaviour. Loss of *endothelin receptor aa* activity leads to higher levels of aggression and less social interaction when shoaling. *ednraa*^−/−^ also have a lower level of whole body cortisol than WT zebrafish. Together, these results suggest that *ednraa*^−/−^ fish might display a dominant behavioural phenotype^33,37^. However, further information would be required to confirm this suggestion – for example by measuring the level of 11-ketotestosterone in this mutant line^43,44^. Pharmacological manipulation of both AVP and 5-HT can rescue the decreased sociality of *ednraa*^−/−^ providing insights into the mechanism underlying the decrease in social behaviour.

The most striking phenotype shown by *ednraa*^−/−^ is a strong decrease in social interaction from juvenile stages up to adulthood, manifested as an increase in both the nearest neighbour distance and inter-individual distance (Fig. 1). This decreased sociality is not dependent upon the number of individuals in a group (6 vs 16, Fig. 1g–j) but is affected by the size of the test arena or the amount of time spent interacting (Fig. 1d,e,g,h). This suggests that mutants maximise their social spacing within the constraints of our laboratory setup. In agreement with this, *ednraa*^−/−^ display a Clark-Evans index of 1.59 indicating that they avoid each other^34^. Conversely, the ratio of 0.61 shown by WT indicates social interaction typical of shoaling. In the mixed genotype shoal, both the inter-individual distance and nearest neighbour distances were larger than in groups of WT fish alone (Fig. 2a–d), and there was an increase in variance of the Clark-Evans index as *ednraa*^−/−^ fish were added (Fig. 2e–h). However, the fish used in this experiment still formed a single group, meaning that it was not possible to separate WT and *ednraa*^−/−^ on the basis of their behaviour. This means that introduction of some mutants was sufficient to alter group cohesion. Furthermore, in the social preference test *ednraa*^−/−^ spent more time interacting with (and being aggressive towards) mutants rather than WT (Fig. 3c). This suggests that the stimulus fish can alter the behaviour of the focal fish in this test, perhaps by aggressive display towards it. Aggression may thus be a determinant of some social interactions in zebrafish.

Although mutants were less social across their lifespan, only adult *ednraa*^−/−^ mutants displayed heightened aggression in a mirror test. This could mean that the aggression and shoaling phenotype are separate entities, or that there is a different basis of social behaviour in juvenile and adult fish. For example, the neural circuits that control shoaling may mature earlier than those that underpin aggression permitting the social phenotype to be expressed more precociously^45,46^.

Aggressive interactions can determine position in a social hierarchy. For example, zebrafish that are consistently more successful in agonistic contests tend to be dominant^33,37,47^. Animals can use either individual recognition or the presence of a status signal to assess social status^48^. However, analysis of the mixed genotype shoal only lasted for ten minutes, decreasing the likelihood of social hierarchy formation^33,37^. This suggests that *ednraa*^−/−^ fish may use an unknown status signal to advertise their aggression. Although *ednraa*^−/−^ larvae transiently express ectopic melanocytes^49^, adult mutants show a similar pigmentation pattern as WT. Moreover, both genotypes are of a similar length (WT 3.46 ± 0.06 cm, *ednraa*^−/−^ 3.59 ± 0.06 cm) suggesting that neither colour nor size form the basis of this signal.

Mutation of *ednraa* alters the distribution of AVP neurons in the preoptic area (POA). At 6 and 12 days of development, the expression of *avp* in the preoptic area was similar in WT and *ednraa*^−/−^ larvae (Fig. 6). However, adult mutants have larger magnocellular AVP neurons, fewer parvocellular AVP neurons and a global decrease of AVP levels in the brain. This suggests that at early stages mutants generate a similar number of *avp*-positive neurons, but they are not maintained during juvenile development. In contrast, there is no effect on the number of POA neurons expressing the related nonapeptide OXT. ETs are co-expressed with AVP in magnocellular neurons of the paraventricular and supraoptic nuclei (mammalian homologues of the POA^12,50^). Systemic and central application of ET can modify AVP secretion^9,51^. Similarly, AVP can also stimulate ET-1 production demonstrating extensive connections between these neurotransmitter systems^51^.

AVP and its receptors are expressed in areas of the vertebrate brain that are important for aggression and social behaviour including nodes of the social decision making network^7,52–55^. Magnocellular AVP neurons project to the dorsal motor nucleus of the vagus^56^ as well as innervating the autonomic nervous system^57^. They also release AVP into the bloodstream via the posterior pituitary. AVP produced in the parvocellular POA is transported to the anterior pituitary where it can potentiate the release of adrenocorticotropic hormone and cortisol in conjunction with other signals as part of hypothalamic-pituitary-adrenal axis^58^. The reduction of parvocellular AVP neurons may lead to the reduction in cortisol levels observed in *ednraa*^−/−^ fish.

AVP has been linked to aggression, dominance and social behaviour in many vertebrate species^54,59^. Dominant zebrafish have more magnocellular AVP neurons and fewer parvocellular AVP neurons^37^ in agreement with our *ednraa*^−/−^ data, in which mutants have larger dorsal AVP neurons than WT (WT mean area: 98.05 ± 12.66 µm^2^; *ednraa*^−/−^ mean area: 182.5 ± 12.19 µm^2^. Student t-test: t_(4)_ = 4.804, p = 0.0086). The concentration of AVP in the brain has also been shown to correlate with sociality. AVP levels are lower in the brains of dominant fish of several species^60,61^ in agreement with our *ednraa*^−/−^ data (Fig. 6k). Manipulation of AVP can also modify aggression^62–64^. Injecting AVP into WT zebrafish increases social interaction and decreases both aggression and fear of a predator^33,41^ and AVP injection rescued the social phenotype of *ednraa*^−/−^ (Fig. 9a). Conversely, AVP *inhibits* social interaction in goldfish demonstrating the varied function of this neurotransmitter across species^65,66^. AVP has also been linked to social recognition^67^. Decreased release of AVP is accompanied by reduced social recognition in naturally occurring *Avp*^−/−^ Brattleboro rats^68,69^ and intranasal AVP application improves social familiarity in humans^70^. This suggests that the reduction of social behaviour in *ednraa*^−/−^ zebrafish could be due to an alteration in the apparent valence or salience of the stimulus fish.

Loss of *ednraa* function increased the basal levels of dopamine, 5-HT and 5HIAA in the brain (Fig. 8). ETs have been shown to modulate dopamine synthesis in other species by altering *Tyrosine hydroxylase* mRNA expression and phosphorylating the Th protein^30,71,72^. In agreement with this, qPCR analysis of *ednraa*^−/−^ mutants identified an upregulation of *th1*, *th2*, *tph1a*, *tph1b* and *tph2* gene expression in the brain (Fig. 7d) suggesting synthesis of dopamine and 5-HT is heightened. Dopamine has a prosocial role in zebrafish in keeping with the role of this neurotransmitter in the reward pathway. There is a positive correlation between the concentration of dopamine and 5-HT and the development of shoaling^73,74^. Furthermore, treatment of zebrafish with the D1 receptor antagonist SCH23390 reduces social preference^75^. 5-HT may not directly control social behaviour but it does modulate aggression and anxiety^33,76–78^, both of which can affect the decision to shoal^79^. The heightened levels of monoamines in *ednraa*^−/−^ do not agree with previous studies in which the maturation of sociality correlates with heightened dopamine and 5-HT levels^73,74^. This might be explained by compensation by other signalling pathways or the combined imbalance of both neurotransmitters. Alternatively, in the absence of *ednraa*, the activity of the other zebrafish ET receptor orthologues (*ednrab*, *ednrba* or *ednrbb*) might be upregulated.

ET and AVP are also potent vasoconstrictors^80,81^. ET receptors are expressed in blood vessels and arterial baroreceptors, where they are involved in the control of blood pressure, heart rate and sodium homeostasis^9^. Similarly, both AVP and cortisol can modify water balance, blood pressure and cardiac output^82^. AVP neurons can be activated by osmotic stimulation showing crosstalk between the autonomic and central nervous systems^82^. As well as acting within the brain to alter social behaviour, the reduction of ET signalling in *ednraa*^−/−^ might impact upon whole-body physiology, including blood flow to the brain and periphery or water balance. Any changes to these homeostatic systems in *ednraa*^−/−^, and their possible contribution to the social behaviour phenotype, will be the focus of future studies.

In humans, plasma ET levels are associated with stress reactivity, socio-economic status and perceived ethnic discrimination^83^. Mutations in *ENDOTHELIN RECEPTOR TYPE B*, *ENDOTHELIN CONVERTING ENZYMES 1* and *2* and *G-PROTEIN-COUPLED RECEPTOR 37* (which codes for a protein homologous to ET-A and ET-B) are linked to autism spectrum disorder^21,22^. In addition, variants in the *AVPR1A* promoter are weakly linked to autism^23^, lower AVP levels correlate with structural brain alterations in autistic patients^24^ and there is a positive association between blood AVP concentration and theory of mind ability in autistic children^25^. In this study we have shown that reduced *ednraa* activity triggers decreased sociality and a reduction in basal cortisol levels. Autism patients also show abnormal regulation of the hypothalamus-pituitary-adrenal axis suggesting that stress response may play an important role in this disease^84^. Zebrafish *ednraa*^−/−^ mutants represent an excellent model to explore the significance of ET signalling for social behaviour, aggression and dominance, with the potential to provide insights into human psychiatric disorders that include changes in sociality.

## Materials and Methods

### Zebrafish strains, care and maintenance

Adult zebrafish were maintained at the University of Leicester using standard protocols and in accordance with institute guidelines for animal welfare. All work was conducted under a UK Home Office licence and was approved by a local Animal Welfare and Ethical Review Body (AWERB) committee. The following strains were used: AB wild-type and *pde*^*tj262/tj262*^ mutants (created in the AB background) that harbour a mutation in *endothelin receptor type aa*^49^, here on referred to as *ednraa*^−/−^. The *pde*^*tj262*^ allele contains a deletion of exon 7 predicted to cause a frame shift in exon 8^85^. The following primers were used to identify mutant fish: forward, atggccattacgacgctaca; reverse, ccaagcacaaggccttttag, with an expected amplicon of 1350 bp for WT and 1220 bp for *ednraa*^−/−^.

### Behavioural methods

Juvenile (one month-old) and adult zebrafish (between 12–18 months of age) were size matched before behavioural analysis. Both males and females were recorded, with no sex difference in behaviour observed. Juvenile fish of both genotypes were of a similar size (WT, 7.73 ± 0.02 mm; *ednraa*^−/−^, 7.73 ± 0.01 mm). Adult fish of both genotypes were of a similar length (WT, 3.46 ± 0.06 cm; *ednraa*^−/−^, 3.59 ± 0.06 cm). Experiments were performed in a dedicated behavioural room under constant illumination and temperature. Behaviour was recorded between 11:00 and 17:00. Zebrafish were transported to the testing room on the same day as the experiments and were allowed to habituate for 1 h. Behavioural experiments were performed using FlyCapture2 2.5.2.3 software and a digital camera from Point Grey Research. Ethovision XT 8 (Noldus) and VpCore2 (ViewPoint Life Sciences) software was used for video tracking of single or groups of fish respectively. To remove observer bias in manual quantification aggression was analysed by two independent researchers blind to the genotype or treatment being analysed. Aggression was scored as the time spent biting or pushing against the mirror and thrashing the tail fin^86^.

### Novel tank diving test

Anxiety-like behaviour and exploratory activity were measured in the novel tank diving test using a standard 1.5 L trapezoid tank^36^. Single fish were placed into this setup for 5 min. We measured the amount of time spent in the bottom (geotaxis), middle and top third of the tank, total distance swum, time spent freezing and mean absolute angular velocity (the frequency of turns made when swimming).

### Open field test

The open field test was performed in an open tank (43 × 22 cm) with opaque walls covered externally with a white material to reduce reflection. The tank was filled with 8 cm of water. Single fish were recorded from above in a 5 min trial in which we measured total distance swum, the duration of thigmotaxis (time spent swimming at a distance of 2 cm or less from the walls), time spent in the centre of the tank (equivalent to half of the total tank area), time spent freezing and mean absolute angular velocity.

### Novel-object boldness

Novel-object boldness was measured using the setup described in^77^. The tank walls were covered with a white opaque material as described above. The object was a 15 ml Falcon tube filled with dark blue and yellow modelling clay suspended midway in the water column at one end of the tank. Single fish were placed into the setup and the time spent within one body length of the novel object was recorded.

### Shoaling

Shoaling was measured in plastic tanks measuring either 12 × 6 cm (small tank, 4 cm water depth) for one month-old juveniles, 43 × 22 cm (medium tank, 8 cm water depth) or 80 × 40 cm (large tank, 10 cm water depth) for adults. Groups of familiar fish were placed in the tank, left to acclimatise, and filmed from above as described in^87^. One-month old juveniles were filmed in groups of 6. They were left to acclimatise for 5 min and filmed from above for 10 min. Adult zebrafish were analysed in groups of either 6 (medium tank, 5 min acclimatisation, 10 minute recording) or 16 (large tank, 24 h acclimatisation, 20 min recording). Groups of 16 fish were given 24 h to habituate to the larger novel arena. We used VpCore2 software (ViewPoint Life Sciences) to track the fish and measure the average nearest neighbour and inter-individual distances. For the mixed-genotype experiment, age- and size matched WT and *ednraa*^−/−^ were allowed to interact for 5 min before recording their behaviour. We compared the average nearest neighbour and inter-individual distances for groups of 6 WT fish, 1 WT and 6 *ednraa*^−/−^, 3 WT and 3 *ednraa*^−/−^ and 6 *ednraa*^−/−^.

### Clark-Evans index

The Clark-Evans index *R* has been shown to gives a measure of the clustering of a number of group of individuals in behavioural studies^88,89^. It is calculated as:

$$\begin{array}{ccc}{\bar{r}}_{A}=\frac{\sum r}{N} & {\bar{r}}_{E}=\frac{1}{2\sqrt{\rho }} & R=\frac{{\bar{r}}_{A}}{{\bar{r}}_{E}}\end{array}$$

r = Distance from a given individual to its nearest neighbour. N = Total number of individuals in the sample. Ρ = Density of random Poisson point process (equal to N).

It is the ratio of the mean nearest neighbour distance (NND) in the observed points (${\bar{r}}_{A}$) to the mean nearest neighbour distance in a random Poisson point process (${\bar{r}}_{E}$). *R* > 1 indicates a greater NND than a random distribution (repulsion), and conversely *R* < 1 indicates a smaller NND than random (aggregation).

R is calculated once per frame for a total of 18,000 measurements per video. The significance of R was tested in each frame using the formula:

$$c=\frac{{\bar{r}}_{A}-{\bar{r}}_{E}\,}{{\sigma }_{{\bar{r}}_{E}}}$$

${\sigma }_{{\bar{r}}_{E}}$ = Standard error of mean nearest neighbour in a random population of the same size as the sample population.

Here, *c* is the standard variation of the normal curve which is then compared to a normal distribution in order to determine significance. The null hypothesis of this test was that animals followed a fully random distribution. R and its significance was calculated using the function *clarkevans*.*test* within the R package *spatstat*^90^.

Frames where p < 0.05 and R > 1 were thus classified as showing significant repulsion, and frames where p < 0.05 and R < 1 were classified as showing significant aggregation. From this, for each video we then determined the proportion of all frames which show significant repulsion, and which show significant attraction.

### Social preference test

The social preference test was adapted from Crawley’s preference for social novelty test for mice^91^ and similar tests in zebrafish^46,92^. We used a transparent plastic tank divided in five compartments: a central area (13 × 19 cm) surrounded by two smaller compartments (6.5 × 9 cm) on either side. The walls between the central and the side compartments contained 1 mm holes to permit movement of water. A single focal fish was placed in the central area and allowed to interact with a stimulus fish placed into the side compartments. The central arena was divided conceptually into four equal size sections (Fig. 3a), and the time the focal fish spent in each area was recorded. We performed two experiments using this setup:

#### Social interaction

This test consisted of two consecutive 5 min recordings. In the first session (interaction 1), an unfamiliar female WT (stranger 1) was placed into one of the small compartments and the focal fish was placed into the central arena. In the second session (interaction 2), a second unfamiliar female WT (stranger 2) was placed in the compartment diagonally-opposite to stranger 1. The choice of compartment to use in each test was randomised. The focal fish was recorded for another 5 min. In interaction 1 we compared the time spent in the central quadrant closest to stranger 1 with the time spent in the central quadrant closest to the empty compartment diagonally opposite. In interaction 2 we compared the time spent near stranger 1 with the time spent near stranger 2. We used females as stimulus fish since they have been found to attract both male and female zebrafish, whereas males induce different responses in males and females^93^. We used 16 WT focal fish (8 males, 8 females; size: 3.37 ± 0.16 cm) and 16 *ednraa*^−/−^ focal fish (8 males, 8 females; size: 3.43 ± 0.03 cm). We used different stimulus fish for each interaction (size: 3.36 ± 0.07 cm).

#### Social discrimination

In this test we placed an unfamiliar female WT (WT stranger) in one compartment and an unfamiliar female mutant (*ednraa*^−/−^ stranger) in the compartment diagonally-opposite. We assessed the preference of WT and *ednraa*^−/−^ focal fish (both male and female) when stranger fish of each genotype were presented simultaneously in a 5 min recording. The time spent in the proximity of each stranger was measured. We used 16 WT focal fish (8 males, 8 females; size: 3.39 ± 0.13 cm) and 16 *ednraa*^−/−^ focal fish (8 males, 8 females; size: 3.41 ± 0.06 cm). We used different stimulus fish for each focal fish (WT size: 3.40 ± 0.11 cm; *ednraa*^−/−^ size: 3.38 ± 0.14 cm).

### Aggression

One-month old juvenile fish were quantified as previously described^45^. Juvenile fish were placed into small plastic tanks (9 × 4.2 × 4 cm) and recorded from the top for 5 min. Locomotor activity and aggressive display were automatically quantified and expressed as locomotion units and aggression units^45^. We used 34 WT and 35 *ednraa*^−/−^ fish. Adult aggression was measured using mirror-induced stimulation as described in^77^. Single fish were recorded for 5 min from above. The time spent in aggressive display, biting the mirror, thrashing the tail and extending the pectoral fins, was quantified manually using LabWatcher software (ViewPoint Life Sciences). The observer was blind to the genotype of the fish being scored.

### Drug administration

[Arg8]-Vasotocin acetate salt (the non-mammalian homologue of AVP) was purchased from Alfa Aesar (Cat. no. J66551) and buspirone hydrochloride was purchased from Tocris (Cat. no. 0962). AVP was injected intraperitoneally 10 min before shoaling was measured. Body weight was measured to calculate the amount to inject. We injected 5 μg/gbw AVP dissolved in 0.9% saline (Oxoid, Cat. no. BO0334C) using a Hamilton syringe (Sigma, Cat. no. 80200). This concentration was chosen according to^33^. Control animals were given a sham injection of saline before behaviour was measured. Buspirone was applied by acute immersion in system water containing 25 µM drug for 1 h. The concentration was chosen according to previous studies^94^.

### *In situ* hybridisation

*In situ* hybridisation for *arginine vasopressin* (*avp*^95^) and *oxytocin* (*oxt*^95^) was performed according to^96^. Sections were photographed using an optical microscope (GXM L3200B, GT Vision) and ImageFocus 4 software (Euromex Microscopen BV) and figures were assembled in Adobe Photoshop version CS2 (Adobe systems). AVP-positive neurons were counted on 100 µm thick coronal sections of the adult brain using ImageJ software. Cell numbers were compiled in Excel and analysed in Graphpad Prism. We counted both the magnocellular and parvocellular AVP neurons on the basis of blue *in situ* staining.

### AVT immunohistochemistry

The anti-AVT antibody was a generous gift from Dr Soojin Ryu (Johannes Gutenberg University, Mainz, Germany). Immunofluorescence labelling was carried out according to standard procedures. Brains were dissected fresh and fixed in 4% PFA for 2 days at 4 °C, and were then washed in phosphate buffered saline (PBS) and stored in methanol at −20 °C until processing. 100 μm coronal sections were collected using a vibratome (Leica VT1000 S, Leica Biosystems). After blocking in PBS containing 5% normal goat serum (Sigma, Cat. no. G9023), 1% dimethyl sulfoxide (Sigma, Cat. no. 276855) and 0.2% Triton X-100 (Fisher, Cat. no. 10254640), we incubated in primary antibody (Rabbit anti-AVT, 1:500^38^) for 24 h at 4 °C. The secondary antibody (Goat anti-rabbit Cy5, 1:500; Invitrogen, Cat no. A10523) was incubated for 2 h at room temperature. Brain sections were imaged at the level of the preoptic area using an Olympus FV1000 confocal microscope with a 20x Nikon objective. Images were assembled using Amira software (Thermo Scientific).

### Cell counts and size measurement

Cell counts. We counted all cells labelled by *avp* mRNA in the preoptic area of 5 WT and 6 *ednraa*^−/−^ by comparing sections with same orientation. There were fewer cells in the ventral POA of mutants suggesting a reduction of *avp* expression in parvocellular neurons. Cell size measurements. The size of larger, dorsal magnocellular neurons labelled by AVP antibody was quantified by measuring their diameters in ImageJ. We measured n = 3 brains for both WT and *ednraa*^−/−^. We counted 12 cells in WT1, 11 cells in WT 2 and 13 cells in WT3. Correspondingly, we measured 8 cells in *ednraa*^−/−^ 1, 10 cells in *ednraa*^−/−^ 2 and 12 cells in *ednraa*^−/−^ 3.

### Real-time quantitative PCR

Primers for *avp* were designed and optimised by Primerdesign Ltd. The primer sequences were: *avp* forward: 5′-CTGCCTGCTACATCCAGAACT-3′, *avp* reverse: 5′-CACACGACATACACTGTCTGATG-3′. The sequences of the primers for *oxt*, *th*, *th2*, *tph1a*, *tph1b*, *tph2*, *avpr1aa*, *avpr1ab* were taken from^33^ and purchased from Sigma. RNA was extracted from the whole brain using the GeneElute^TM^ Mammalian Total RNA Miniprep Kit (Sigma-Aldrich) followed by a DNase treatment with Turbo DNase (Ambion). The quality and quantity of RNA was assessed using a Nanodrop 2000 (Thermo Scientific). cDNA was synthesised from 0.5 µg of RNA using the RevertAid First Strand cDNA Synthesis Kit (Thermo Scientific). Real-time PCR was performed on 8 whole brains per genotype with three replicates for each brain using a CFX Connect^TM^ Real-Time System machine (BIORAD) and the SensiFAST^TM^ SYBR No-ROX Mix (Bioline). The PCR conditions were 95 °C for 2 min followed by 40 cycles of 95 °C for 15 s, 60 °C for 15 s and 72 °C for 30 s. Results were normalised to the expression level of the housekeeping gene *rpl13*. The relative expression of the genes was calculated using the comparative 2^−ΔΔCt^ method as described in^97^.

### Enzyme-linked immunosorbant assay (ELISA) for AVP

We used an Arginine Vasopressin ELISA kit that is 100% specific for both AVT and AVP to measure the basal levels of AVP (Cayman, Cat. No. 583951). The extraction and purification of AVP was carried out as previously described^98,99^. Nine WT and 7 *ednraa*^−/−^ brains were dissected, snap frozen in liquid nitrogen and stored at −80 °C. Brain areas were weighed and homogenised in 1 ml H_2_O acidified with 3 μL glacial acetic acid (Fisher, Cat. No. 10394970) using a glass pestle and mortar. They were placed into a boiling water bath for 3.5 min. The homogenates were centrifuged at 12,000 × g for 20 min at 4 °C. The supernatants were loaded onto solid phase extraction (SPE) columns (HyperSep C18 100 mg/1 ml; Thermo Scientific, Cat. No. 60108-302) conditioned with 3 ml methanol and 3 ml H_2_O. To purify the samples, columns were washed sequentially with 1 ml 5% acetic acid, 1 ml H_2_O and 1 ml 5% methanol. Peptides were eluted with 2 ml ethanol:6 M HCl (2000:1 v/v). The eluate was dried by evaporation and was recovered in 100 µl EIA buffer (provided in the EIA kit). The assay was performed on two replicates of each sample according to the manufacturer’s instructions. Absorbance values were read on a plate reader (iMark^TM^ BIO-RAD). The concentration of AVP in the samples was calculated using the EIADouble Excel workbook provided by Cayman (www.caymanchem.com/analysisTools/elisa).

### ELISA for cortisol

To measure the basal levels of cortisol a total of 11 wild-type and 12 *ednraa*^−/−^ were flash frozen in liquid nitrogen and stored at −80 °C. Whole body cortisol extraction and the ELISA assay were performed according to^100^ with minor modifications. The fish were thawed, the head removed and single bodies homogenised in 2 ml microcentrifuge tubes (Eppendorf) in 1 ml of ice cold PBS using a Ultra Turrax T8 Homogenizer (IKA). The extraction was carried out using ethyl-acetate (Fisher). ELISA was performed using the human salivary cortisol kit (Salimetrics) and results were recorded using a plate reader.

### High performance liquid chromatography (HPLC) analysis of monoamines and their metabolites

HPLC was performed on 7–9 brain regions of each genotype. The brain was divided into olfactory bulb, telencephalon, diencephalon, optic tectum, cerebellum and medulla at room temperature under a microscope. Samples were weighed, homogenised in 100 µl ice-cold 0.1 N perchloric acid and centrifuged. HPLC with electrochemical detection was used to measure dopamine, serotonin (5-HT), 3,4-dihydroxyphenylacetic acid (DOPAC), homovanillic acid (HVA) and 5-hydroxyindoleacetic acid (5-HIAA). Samples were compared to standard solutions of known concentrations and the results were expressed as fmol/mg of brain.

### Statistical analysis

Data are presented as scatter plots, bar charts or line graphs showing the mean and the standard error of the mean (SEM). Each dot represents an individual fish from one experiment. Data were assessed for normality using D’Agostino & Pearson normality test. The equality of variances was tested using an F-test. We used unpaired Student’s t-tests (with Welch’s correction if appropriate), Mann Whitney U tests. One-way ANOVA followed by Tukey’s post hoc and two-way ANOVA followed by Tukey’s post hoc (for significant interaction between factors) or Sidaks’ post hoc (for non-significant interactions between factors) was used for multiple group comparisons. Data were collected in Excel (Microsoft) and statistical analyses were carried out with GraphPad Prism7. For individual tracking, groups of fish were analysed using the idTracker software^101^. The Clark-Evans aggregation index (*R*) was calculated for each frame in the resulting tracks^34^. The result is a measure of the clustering of the animals in each frame, calculated as the ratio of the mean nearest neighbour distance in that frame to that expected for a Poisson point process of the same intensity. *R* < 1 suggests aggregation, whilst *R* > 1 suggests repulsion. Analyses were carried out in R version 3.4.3 (R Core Team, 2017) using package “spatstat”^102^. Statistical significance was depicted as follows: *p < 0.05, **p < 0.01, ***p < 0.001, ****p < 0.0001. For multiple comparisons, letters not shared in common between or amongst groups in figure graphs indicate significant differences.

## Supplementary information

Clark-Evans index

Shoaling behaviour of 16 WT fish in a large tank

Shoaling behaviour of 16 <i>ednraa<sup>-/-</sup></i> in a large tank
