# Supplementary material for: Endothelin neurotransmitter signalling controls zebrafish social behaviour
Source: Sci Rep. 2019 Feb 28;9:3040. doi: 10.1038/s41598-019-39907-7 (PMC6395658; doi:10.1038/s41598-019-39907-7)
Supplement: Supplementary file 1 — Clark-Evans index [file 41598_2019_39907_MOESM1_ESM.pdf]

Endothelin neurotransmitter signalling controls zebrafish social behaviour

Héctor Carreño Gutiérrez[1], Sarah Colanesi[2], Ben Cooper[1], Florian Reichmann[1], Andrew M.J. Young[1], Robert N. Kelsh[2] and William H.J. Norton[*1].

1. Department of Neuroscience, Psychology and Behaviour, College of Life Sciences, University of Leicester, Leicester, LE1 7RH, UK.

2. Department of Biology and Biochemistry and Centre for Regenerative Medicine, University of Bath, Claverton Down, Bath, BA2 7AY, UK.

[*]Corresponding author: **T:** +44 (0)116 252 5078, **E:** whjn1@le.ac.uk

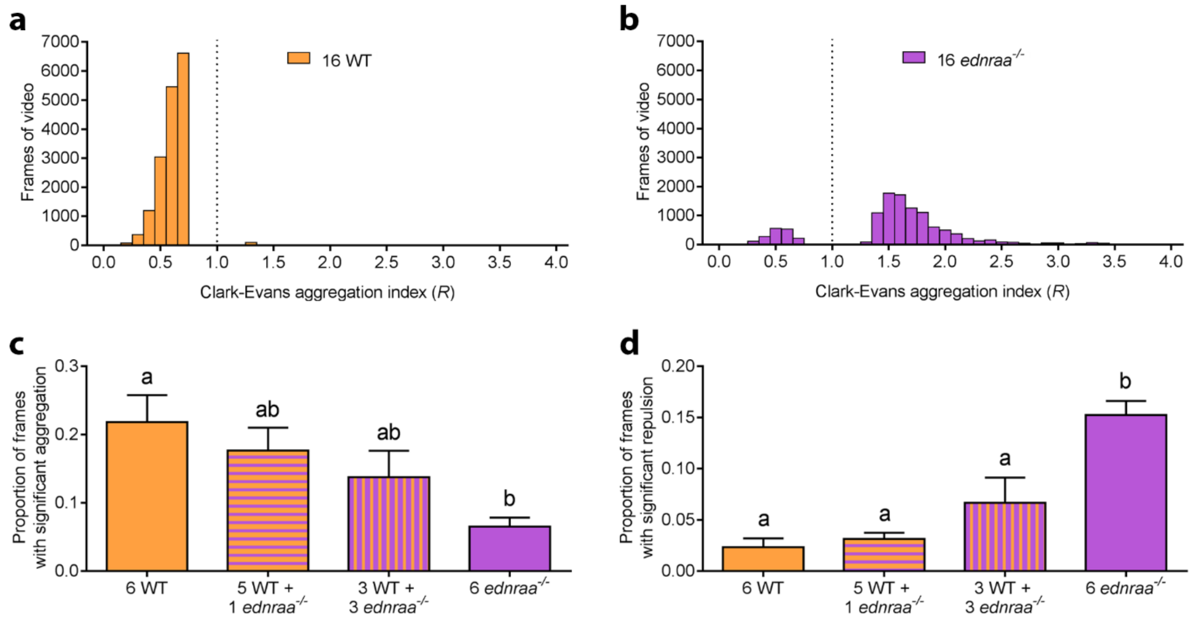

Supplemental figure 1. Clark-Evans index. (a,b) Shoaling test in a large tank. **(a)** A shoal of 16 WT fish show less repulsion and more attraction to one another compared to **(b)** a shoal of 16 *ednraa*$^{-/-}$ fish, as measured with the Clark-Evans aggregation index *R*. Only frames in which aggregation or repulsion were significant ($p<0.05$) are shown here. **(c,d)** Shoaling test of mixed genotypes. As the proportion of *ednraa*$^{-/-}$ in a group of 6 fish increases, there tend to be fewer frames showing significant aggregation **(c)** and more frames showing significant repulsion **(d)** measured with the Clark-Evans aggregation index *R*. Letters not shared in common between or amongst groups indicate significant differences from Tukey's post hoc comparisons after one-way ANOVA, $p <0.05$.

Film 1. Shoaling behaviour of 16 WT fish in a large tank.

Film 2. Shoaling behaviour of 16 *ednraa*$^{-/-}$ in a large tank.
